# Loss-of-heterozygosity facilitates a fitness valley crossing in experimentally evolved multicellular yeast

**DOI:** 10.1098/rspb.2021.2722

**Published:** 2022-06-08

**Authors:** Beatriz Baselga-Cervera, Noah Gettle, Michael Travisano

**Affiliations:** ^1^ Department of Ecology, Evolution, and Behavior, University of Minnesota, St Paul, MN 55108, USA; ^2^ The BioTechnology Institute, University of Minnesota, St Paul, MN 55108, USA; ^3^ Minnesota Center for Philosophy of Science, University of Minnesota, Minneapolis, MN 55455, USA; ^4^ Department of Zoology, Stockholm University, Stockholm, Sweden

**Keywords:** multicellularity, underdominance, evolutionary landscapes, genotype–phenotype map, loss-of-heterozygosity, loss of function

## Abstract

Determining how adaptive possibilities do or do not become evolutionary realities is central to understanding the tempo and mode of evolutionary change. Some of the simplest evolutionary landscapes arise from underdominance at a single locus where the fitness valley consists of only one less-fit genotype. Despite their potential for rapid evolutionary change, few such examples have been investigated. We capitalized on an experimental system in which a significant evolutionary shift, the transition from uni-to-multicellularity, was observed in asexual diploid populations of *Saccharomyces cerevisiae* experimentally selected for increased settling rates. The multicellular phenotype results from recessive single-locus mutations that undergo loss-of-heterozygosity (LOH) events. By reconstructing the necessary heterozygous intermediate steps, we found that the evolution of multicellularity involves a decrease in size during the first steps. Heterozygous genotypes are 20% smaller in size than genotypes with functional alleles. Nevertheless, populations of heterozygotes give rise to multicellular genotypes more readily than unicellular genotypes with two functional alleles, by rapid LOH events. LOH drives adaptation that may enable rapid evolution in diploid yeast. Together these results show discordance between the phenotypic and genotypic multicellular transition. The evolutionary path to multicellularity, and the adaptive benefits of increased size, requires initial size reductions.

## 1. Introduction

Adaptation shapes and reshapes species, generating biological diversity. Recognizing the importance of adaptation, however, leaves open questions about its limits and the identification of factors that either facilitate or impede adaptive evolution. Fitness landscapes provide a valuable way to conceptualize the possible adaptive space in which a population can move [1]. The topography of such landscapes can be thought of in terms of the underlying genetic changes that impact fitness. Highly polygenic traits allow for many possible trajectories in terms of fitness increases or decreases, generating landscapes with numerous broad peaks and valleys. In such cases, crossing valleys between adaptive peaks requires multiple possible steps of reduced fitness, decreasing the likelihood that a population will reach the global optimum [2]. In cases where fewer loci are involved, however, the possibility space for changes in fitness are narrowed, as are fitness valleys, eventually allowing for the increased ability of populations to traverse more of the landscape [3]. Indeed, some of the narrowest fitness valleys, potentially the easiest to cross, arise from underdominance at a single locus since the fitness valley consists of only one less-fit genotype. Unfortunately, few such examples have been investigated or even described despite the potential for rapid and significant evolutionary change on simple fitness landscapes [4,5].

Here, we describe an experimental system in which a major evolutionary shift, the transition from uni- to multicellularity, occurs by single-locus changes. We focus on the heterozygous intermediate state. Previous studies observed the rapid evolution of complex multicellular phenotypes, snowflake yeast, in laboratory populations of diploid yeast *Saccharomyces cerevisiae* when selected for increased settling rates in liquid media [6]. Multicellular individuals consist of tens to hundreds of cells, in which daughter cells fail to separate from their mother cells following cell division. These phenotypic shifts are the product of single-locus loss or partial loss of function (LOF) mutations [6,7], previously identified as recessive [8]. This experimental system represents a major transition [9] with a simple, one-locus fitness landscape.

**Figure 1.**
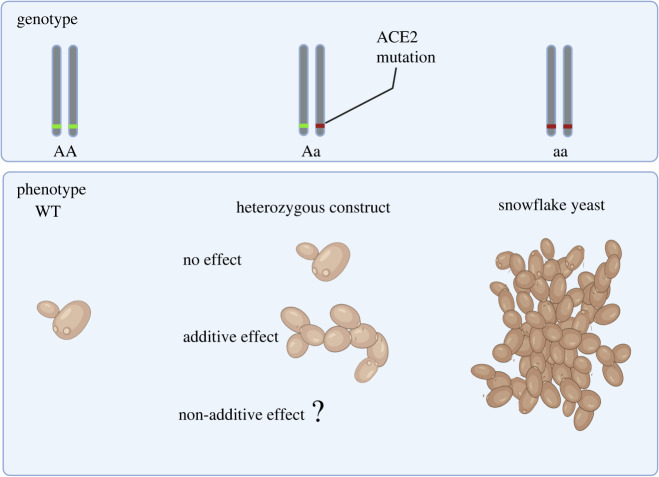
Schematic of genotype–phenotype associations for ACE2 mutations in the *Saccharomyces cerevisiae* strain Y55 (WT) genetic background. Strains homozygous for partial or total LOF mutations in the ACE2 transcription factor generate snowflake yeast multicellular phenotypes. Genotypes heterozygous for ACE2 mutations do not have a multicellular phenotype and were previously described as phenotypically WT (no effect of the mutation). In this study, we formally examined the heterozygous phenotypes to determine if this genotype is associated with a previous undetected intermediate phenotype (additive effect) or other unexpected possibilities (non-additive effect). (Online version in colour.)

To better understand how populations navigate valleys in simple fitness landscapes, we reconstruct the genetic landscape between the ancestral unicellular and derived multicellular phenotypes (figure 1). We confirm that yeast homozygous for LOF alleles at the ACE2 locus are multicellular. Heterozygosity at the ACE2 locus, however, does not confer a partial intermediate form of multicellularity. Instead, we show that ACE2 heterozygosity results in smaller size and reduced settling rates compared to the wild-type (WT) and homozygous recessive, counter to the evolutionary trend towards multicellularity observed in settling selection environments. The underdominance for size shows that this system's evolutionary transition to multicellularity does not progress in a phenotypically stepwise manner. Nevertheless, multicellular yeast evolved readily in populations heterozygous for Ace2-multicellularity-generating mutations, even in the absence of settling selection. Together these results show discordance between phenotypic and genotypic evolutionary transitions.

## 2. Material and methods

### (a) Yeast transformation and culture conditions

The baseline ‘WT' strain of *S. cerevisiae* used was a Y55 isolate carrying a kanMX marker and autodiploidized to be homozygous across all loci [10]. Additionally, we constructed two other strains derived from Y55. One is a heterozygous knockout of the gene ACE2 (ACE2/ace2*Δ*::kanMX, abbreviated here as ACE2/ace2). The other strain is heterozygous for a missense mutation in ACE2 known to result in multicellularity (ACE2/ace2Δ::ace2c.1934T > A + kanMX, abbreviated here as ACE2/ace2*) [7]. Transformations were done using a LiOAc/PEG protocol [11], resulting in heterozygous Ace2. Homozygous recessive snowflake multicellular strains were generated from these heterozygous mutants via separation and autodiploidization of haploid spores. Successful transformation and zygosity were confirmed using PCR.

Clonal populations were started from single colonies. Cultures were grown in 25 × 150 mm glass culture tubes with 10 ml aliquots of media, at 30°C and shaking at 250 r.p.m. unless otherwise indicated. Isolates were maintained in Yeast Peptone Dextrose media (YPD; 1% (v/w) yeast extract, 2% (v/w) peptone, 2% (v/w) D-glucose, pH 5.8). Solid plates were prepared by adding 1.5% agar.

### (b) Cell sizing measurements

For size measurements, single genotype (or isolate) populations were grown overnight in YPD. Aliquots of 100 µl from the overnight populations were transferred into 10 ml of fresh medium. Distributions of particle sizes of Y55, ACE2/ace2 and ACE2/ace2* strains were measured after 24 h growth in YPD using a Beckman-Coulter Counter (Multisizer 4) with a 70 µm diameter aperture probe, measuring particles between 3 and 40 µm in diameter. Particle diameters were characterized based on equivalent spherical Heywood diameter. For each isolate, population measurements were replicated five times. Raw multisizer files (*.#m4) were batched with the R package MultisizerToolkit (https://github.com/gettl008/MultisizerToolkit). Biovolume estimates for each volumetric classification were computed as a function of particle density by volume. Instrument calibration adjustments were performed with standard calibration latex particles of 10 µm diameter (Beckman Couter) recommended for the 70 µm aperture tube.

We used a digital flow cytometer (FlowCam 3.0 Fluid Imaging Technologies) to complement the phenotypic assessment of the populations. We obtained additional information about cell sizes, shapes and mother–daughter(s) stage via individual photomicrographs. Three replicate populations per genotype were measured after 24 h growth in YPD. The 20× objective was used to image particles less than 2 µm in diameter at a 0.030 ml min^−1^ flow rate. The sample volume analysed was dependent on the sample concentration. An initial fixed number of particles per sample was imaged. Images were manually processed to remove artefacts. Phenotypes within the populations were manually classified as single cells, mother–daughter couplets, or chains of cells for the phenotype-size correlations. Size and particle concentration were calibrated with bead solutions of 5, 10 and 20 µm sizes (Fluid Imaging Technologies, Inc.). FlowCam images provide both area-based diameter and equivalent spherical diameter estimations.

### (c) Bioassays

We performed a series of bioassays assessing fitness and settling performance to analyse homozygous and heterozygous ACE2 constructs relative to the WT. To measure settling rates, we suspended well-mixed 2.5 ml samples of overnight YPD cultures in 10 ml acrylic 10 × 10 mm cuvettes at room temperature. Settling was visualized over 3 h via a time-lapse video of the static cultures. Pictures were taken every minute for the first 20 min and every 5 min afterward. This assay serves as a visual proxy of the strains settling performance during the benchtop settling selection (see below).

To measure growth, 5 µl of overnight populations were transferred into 96-well plates in 195 µl of YPD. Growth of the strains was measured by OD600 absorbance reads every 10 min using a Tecan infinite 200pro microplate reader. Growth curves were measured as the average of 24 wells per strain. Growth curve assays were replicated independently three times. Growth was also assessed by counting cells/clusters under an optic microscope using a hemocytometer before inoculation and after 24 h batch growth in 10 ml of YPD.

In addition to measuring settling ability and growth, we also measured Malthusian [12] fitness and competition assays. We propagated WT and heterozygous ACE2 mutant single strains and in competition with an unmarked WT Y55 strain, with and without benchtop settling selection (propagation of the bottom 100 µl of 1.5 ml aliquots settled undisturbed in the bench for 7 min). For each fitness assays, three replicate populations were established with 100 µl of an overnight population of single strains genotypes. One hundred microlitre subsamples were transferred to fresh media every 24 h either with or without settling selection over two cycles of selection. Colony-forming units (CFUs) were measured via dilution plating. Single strains' fitness was calculated in Malthusian parameters (*m*) [13,14]. Competition assays were set up using 100 µl of a 50/50 mix of the focal genotype (heterozygote strains; KanMX marked) and the common competitor (Y55 WT). Five independent competitions per focal genotype and selection treatment were measured. Plates were inoculated immediately after the selection step of the life cycle. CFUs of both total populations and focal strains were measured via dilution plating followed by replica plating on G418 YPD plates. The change of relative proportion of the two competing strains over the three cycles of selection was measured as the differential growth rate (*s*) by timepoint (*t*) [15]:

2.1 $$s=\ln\left[\frac{x_{1}(t)}{x_{2}(t)}\right],$$

where *x*_l_(*t*) and *x*_2_(*t*) represent the relative proportion (or number of CFUs) of the two competing strains.

### (d) Settling selection experiments

Three replicate populations per genotype (WT, ace2/ACE2 and ace2*/ACE2 mutant) were subjected to two different selection treatments, standard daily transfers in YPD and benchtop settling selection (see above). Populations were grown and propagated through eight 24 h growth cycles. Every 2 cycles, subcultures from each replicate population were plated to determine the presence of multicellular phenotypes based on colony morphology (smooth and rugose).

A second experiment doubled the number of replicates per treatment under the same conditions. Results were assessed after 10 cycles of benchtop settling selection. At the end of the experiment, freezer stocks were stored in 25% glycerol at −80°C. To evaluate the population's response based on multicellular phenotypic expression, we analysed each final population with a digital flow cytometer.

## 3. Results

### (a) Phenotype expression of the heterozygous intermediate stage

Heterozygote constructs with either the ACE2/ace2 and ACE2/ace2* were smaller than the WT (table 1) with a 20% reduction in median diameter (K-W tests, χ^2^ = 6.3051, d.f. = 2, *p* < 0.05). The distribution in width or span (D90 – D10)/D50, D10 and D90 refer to the diameter where a per cent of the population's size distribution is smaller in size) of the heterozygous mutant strains was also significantly less than that of the WT (table 1; Fligner–Killeen non-parametric test for homogeneity of variances, χ^2^ = 59901, d.f. = 2, *p* < 0.001).

**Table 1.** Descriptive statistics of population size distributions. Size data were collected with a Coulter Counter and phenotype fractions identified from images obtained with FlowCam imaging microscopy. Data represent values of 3 isolates per strain. Median diameter, median absolute deviation (MAD) and span describe values for the total populations. ^a,b^Within a column, values without a common superscript indicate statistically significant differences (*p* < 0.05).

strain	median diameter ± MAD (µm)	span (distribution width)	phenotype fractions ± s.d. (%)		
			single cells	mother–daughter	chains of cells
Y55 ancestral WT	6.9 ± 1.27^a^	0.50^a^	10.9 ± 3.9	52.8 ± 8.2	36.2 ± 6.4
ACE2/ace2 construct	5.48 ± 0.69^b^	0.34^b^	37.4 ± 2.8	57.8 ± 1.8	4.7 ± 0.6
ACE2/ace2* construct	5.62 ± 0.67^b^	0.33^b^	45.9 ± 6.1	49.7 ± 8.1	4.3 ± 1.9

**Figure 2.**
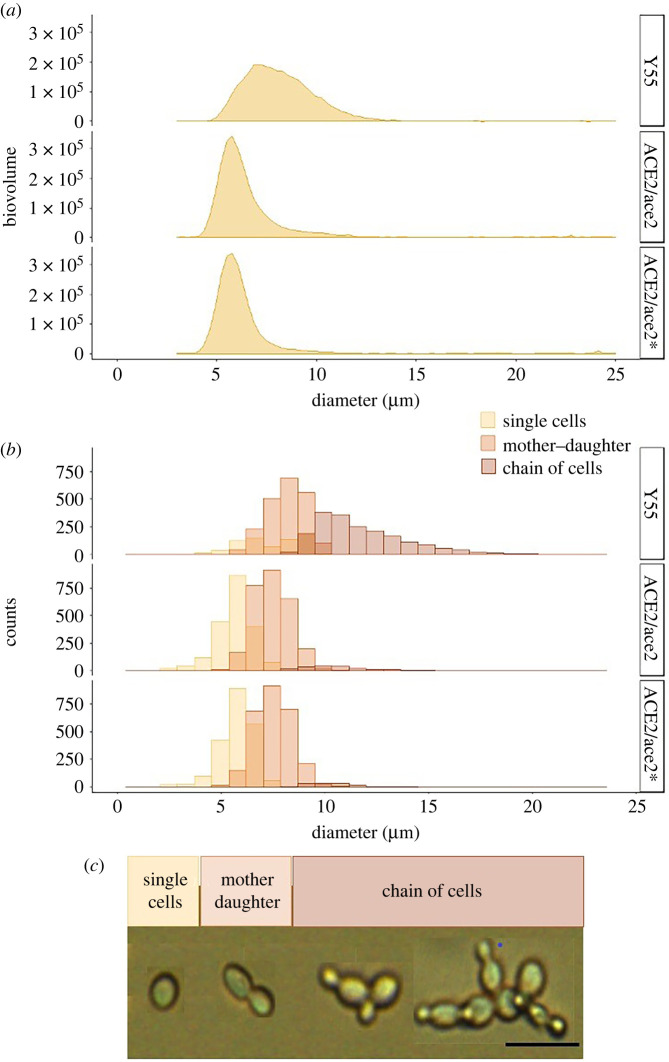
Phenotypic distributions of replicate populations of the ancestral Y55 WT strain and constructed heterozygous strains. (*a*) Population biovolume distributions by diameter (micrometre) of populations of WT and heterozygous ACE2 mutants (Y55, ACE2/ace2 and ACE2/ace2*, respectively). Data obtained with a Coulter Counter Multisizer 4. (*b*) Histograms of the observed cellular phenotypes classes per strain. (*c*) Particle imaging of the cellular phenotypes was classified as single-cells (brown), mother–daughter cells (orange) and chains of more than 2 cells (yellow). Scale bar, 20 µm. Histogram counts and microphotographs were attained with a FlowCam. (Online version in colour.)

In the heterozygotes, the 90th percentile of the population was approximately 6.62 µm diameter, while in the WT it was approximately 8.77 µm diameter. By contrast, WT population diameter distributions were skewed toward larger individuals represented by short chains of cells (figure 2*a*). While both WT and heterozygous populations were composed of similar fractions of mother–daughter pairs, WT populations contained a larger fraction of small chains and significantly fewer single cells (figure 2*b,c*; Pearson's $\chi^{2}=10\;280$, d.f. = 16, *p* < 0.001). Moreover, WT single cells were significantly larger than those of the heterozygous construct (K-W tests comparing single cells diameter per strain, χ^2^ = 1051, d.f. = 2, *p* < 0.001). A more detailed description of the populations is provided in electronic supplementary material, table S1 and figure S1.

### (b) Assessing underdominance in the transitional stage

To determine how an intermediate heterozygous stage might impact transitions from uni- to multicellularity, we examined multiple aspects of fitness associated with the WT and the heterozygous ACE2 constructs. In terms of single strains OD growth rates in YPD, we observed no significant differences (electronic supplementary material, figure S2, Materials). While there was no significant difference in total cell number within populations at 24 h (hemocytometer cellular counts by strain, one-way ANOVA, *F* = 2.48, *p* < 0.5), we found that WT populations had more cells per individual than populations of the heterozygous strains (electronic supplementary material, table S2).

**Figure 3.**
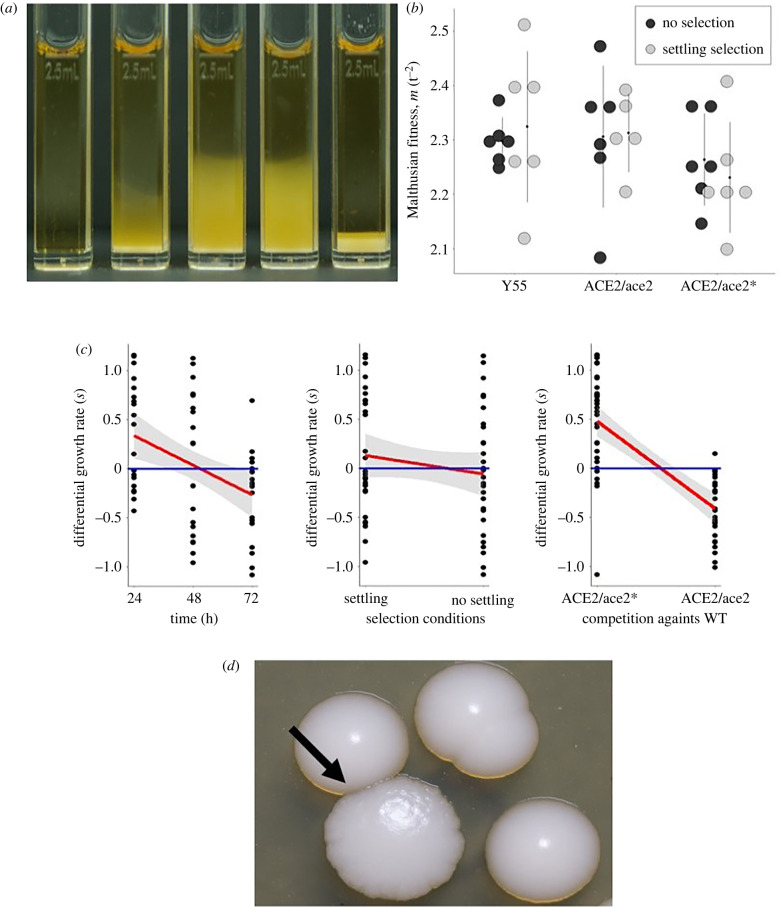
Fitness and growth assays. (*a*) Visual comparison of undisturbed settling of 2.5 ml after 3 h, left to right; negative control, ancestral WT, constructed ACE2/ace2 knockout constructed ACE2/ace2* missense and double knockout ace2/ace2 multicellular strain. (*b*) Malthusian fitness (metre) after two cycles under settling selection and without selection (data from two independent measurements, *p* > 5). (*c*) Linear regression of *s* by time, selection conditions and focal strain in competition against the WT ancestral, over the curse of three cycles of selection. Linear regression analyses indicate a reduction in fitness of the heterozygous strains under settling selection. (*d*) Photograph of a multicellular rugose colony (black arrow), among smooth colonies taken from an initially ACE2 heterozygous population subjected to the settling selection regime. Multicellular colonies were present at the end of all fitness assays involving initially heterozygous constructs (approx. 18 generations). (Online version in colour.)

Settling rates of WT populations in liquid media were much higher than the populations of heterozygous genotypes and significantly worse than populations of the ace2/ace2 homozygous multicellular strain (figure 3*a*; electronic supplementary material, videos S1 and S2). Using single strains Malthusian fitness assays, however, we did not find any significant differences between the WT and heterozygotes in either condition with or without settling selection (figure 3*b*, two-way ANOVA m ∼ strain * selection, *p* > 0.5). The number of CFUs was higher in the heterozygous populations, as supported by the growth assays (electronic supplementary material, figure S3). CFUs account for individuals rather than single cells.

Each heterozygous genotype was competed against the WT ancestral genotype in bench settling and non-settling conditions. The competition's differential growth rate (*s*) over three cycles of selection showed a significant effect of time, selection conditions and heterozygous genotypes (competition) (figure 3*c*; electronic supplementary material, table S3; linear regression analyses). The significant time effect indicates a change in allele frequency. We observed a reduction in fitness of the heterozygous strains under settling conditions when compared to non-settling conditions, indicating that the heterozygous performed worse than the WT under settling conditions given the limits of resolution. The biggest fitness difference observed was between the two heterozygous genotypes. The large effect could be attributed to the founding impact when streaking a single colony to establish the starting single-strain batch culture for the competitions.

### (c) Rapid loss-of-heterozygosity during selection

We observed the formation of multicellular rugose colonies in some of the single strain heterozygous plates and competition plates (figure 3*c* photograph of the rugose colonies). Multicellularity readily evolves based on recessive alleles, but the intermediate heterozygous stage presents the reduced size and settling performance. Contrary to expectations of an undetected intermediate stage, this result suggests the rapid evolution of multicellularity and loss-of-heterozygosity (LOH) events in the heterozygotes. Previous studies have similarly documented LOH events over short evolutionary timescales [16,17].

To assess rates of appearance and turnover of multicellular phenotypes, we propagated three single genotype populations for each of WT and heterozygous ACE2 mutant strains per selection condition (both non-settling and benchtop settling conditions [6]). In non-settling environments, multicellular phenotypes were not detected in any populations after eight transfers. By contrast, in benchtop settling conditions, multicellular individuals were detected by rugose colonies within four transfers (approx. 33 generations) in ancestrally ACE2/ace2 populations, within six growth cycles (approx. 47 generations) for the ancestrally ACE2/ace2* populations but were not detected in the ancestrally WT populations.

**Figure 4.**
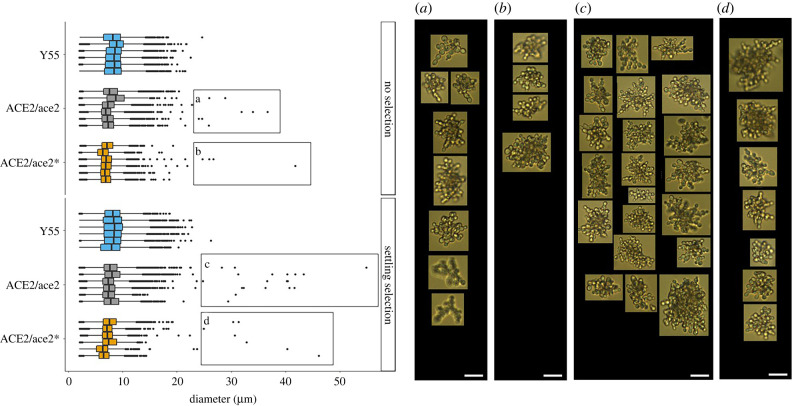
Single-strain replicate populations selected for approximately 70 generations with and without settling selection. Boxplots depict the count's distributions by diameter (micrometre) of 5000 particles imaged per population, under settling selection and without selection. Images were manually checked to eliminate artefacts. Multicellular Snowflake phenotypes were identified and correlated with size. Microphotographs show representative multicellular phenotypes from evolved ACE2/ace2 and ACE2/ace2* populations without selection (*a* and *b*) and under selection (*c* and *d*). Graph points linked with the microphotographs are indicated within squares. Ancestral Y55 WT strains did not evolve multicellular representatives in any treatments, large graph points were manually checked and identified as large chains. Scale bar, 20 µm. (Online version in colour.)

Additionally, we conducted a similar evolution experiment with 6 replicate populations per genotype which were run for 10 growth cycles with and without settling selection (approx. 70 generations) then analysed using a FlowCam. By increasing the number of replicates, we can confirm the occurrence of the LOH events, both in non-settling and benchtop settling conditions, and the effect of selection. Snowflake phenotypes were identified in all ancestrally heterozygous ACE2 mutant ancestors subjected to settling selection (figure 4). In populations that experienced no settling selection, however, multicellular individuals were only identified in 2 out of 6 ACE2/ace2* replicate populations and 4 out of 6 ACE2/ace2 replicate populations (figure 4). No multicellular individuals were found in the ancestrally WT populations propagated in either environment.

## 4. Discussion

Adaptation shapes the diversity of life, but what shapes adaptation remains a topic of intense debate [18]. Advances in predicting adaptive evolution [19] demonstrate progress in delimiting the scope for incremental adaptive improvement, but the prospective determination of the possibilities for adaptation remains difficult [20]. Even retrospective investigations into the basis for adaptation are often contentious [21] and open to frequent reevaluation [22]. The possibilities for adaptation appear to be simultaneously ubiquitous in shaping the diversity of life but also frequently overstated [23]. Long periods of stasis are commonly found in the fossil record [24]. Many transformative adaptations such as the evolution of eukaryotes or complex multicellularity are rare despite the adaptive outcomes they potentiate. Determining how and why adaptive possibilities do or do not become evolutionary realities is central to understanding the tempo of evolutionary change [25].

Previous studies showed that genotypes homozygous for LOF alleles have multicellular phenotypes, and genotypes that are either heterozygous or homozygous for functional alleles [6] are unicellular. This pattern of unicellularity and multicellularity is consistent with the standard expectation of LOF alleles and the theory for allelic dominance [26]. Our current study uncovers surprising phenotypic complexity in the transition to multicellularity, belying the simplicity of a single locus genetic system. Heterozygous genotypes are underdominant for multicellularity and are substantially more unicellular than the WT. In addition, we found reduced settling rates in heterozygotes relative to WT homozygotes, although fitness costs in the settling selection environment were not detected. By contrast, during short-term experimental evolution, multicellular genotypes are more likely to arise in populations of heterozygotes than WT populations. The predisposition of heterozygotes to evolve multicellularity, despite underdominance, demonstrates a discordance between phenotypic and genotypic aspects of adaptive transitions.

### (a) Adaptive novelty mediated by recessive mutations

To better understand the evolutionary path that populations take when selected for settling ability, we reconstructed the genetic landscape between the ancestral unicellular and derived multicellular phenotype. Evolved multicellularity in this system results from single partial or total LOF mutations in one set of genes associated with mother–daughter cell separation followed by a LOH event [27]. Yeast heterozygous for these mutations remain unicellular. We focused on mutational effects on one of these multicellularity-associated genes, ACE2, to better understand the evolutionary transition from uni- to multicellularity in this system.

The heterozygous ACE2 mutations we tested showed signs of a disadvantage in size and settling ability, a key fitness component in the evolution of multicellularity in the original selection experiment [6]. Heterozygotes, however, did not result in statistically significant costs in terms of growth and reproduction. These results suggest that rather than being entirely recessive, these mutations are non-additively underdominant [28]. Selection against the heterozygous state leads to a bistable evolutionary dynamic resembling two peaks separated by a low fitness valley.

Recessiveness and/or underdominance mutations have often been regarded as obstacles to fixation in diploid populations, particularly those that are predominately asexual [29]. Threshold frequencies for recessive genotypes to fix are partially determined by the fitness values of the genotypes involved. Different research approaches addressing have identified low thresholds for recessive gene frequencies fixation in the populations (i.e. studies in multiple loci with weak effects [30] or ‘engineered underdominance' approaches in insect populations for pest control [31,32]). With scenarios of underdominance with large fitness effects, if the population is sufficiently large, the mutant recessive allele could be maintained for several generations [33]. Therefore, we conclude that stable polymorphisms are maintained in our system despite mild underdominance, making the heterozygote genetic state transitory leading to the evolution of multicellularity. LoF mutations represent a genetic variation resource correlated with biodiversity, adaptation and evolutionary novelty [29,34,35].

### (b) Genotype–phenotype mapping: is there continuity?

Both the knockout and missense mutation we introduced into ACE2 resulted in a significantly reduced cell size and individuals with fewer cells when in a heterozygous state than the WT allele. In budding yeasts, the main known function of Ace2p is as a transcription factor, controlling the expression of genes that degrade the secondary septum the last connection between budding mother–daughter pairs. Although ACE2 expression has been correlated with cell size control [36], much less is known about other roles this gene plays in yeast cell physiology. While this study did not directly address the mechanism by which yeast heterozygous for LOF mutations in ACE2 result in these traits, it appears that it may play a bigger role in cell division and cell cycle than previously recognized. Moreover, this role only becomes clear when this gene is examined in light of dosage rather than presence or absence.

Organisms heterozygous for LOF mutations are often not phenotypically distinguishable from the WT [37]. Moreover, screens in *S. cerevisiae* suggest that only 3% of approximately 5900 yeast heterozygous deletion mutations result in detectible phenotypic change [37]. In the case of ACE2, previous studies in *Candida albicans* have shown that heterozygous knockouts are not phenotypically discernible from WT. [38]. The extent of phenotypic assessment in these types of studies, however, generally only happens in single environments. By contrast, we identified simple haploinsufficiency in ACE2 LOF heterozygotes that result in measurable phenotypic differences. While our system may represent an outlier with respect to the phenotypic effects of heterozygosity in yeast, the importance of environmental context with regards to heterozygosity-specific expression has been previously shown elsewhere [39]. Indeed, preliminary results suggest that the phenotypic differences between ACE2 LOF heterozygotes and WT largely disappear when these strains are grown in minimal rather than rich media.

Major transitions in evolution, such as the origin of multicellularity, are often regarded as a series of minor steps [40]. For instance, comparative biology has largely been successful in reconstructing a stepwise program for the evolution of multicellularity in volvocine algae [41]. Intermediate forms towards multicellularity can be mapped based on the appearance of novel features in an additive gradient. Thus, one might expect continuity of morphological characters across most such transitions. However, we did not find such an evolutionary gradient in the experimental evolution of multicellular yeast. Rather there is only one intermediate genotypic state between ancestral unicellularity and relatively complex multicellularity in our study. This heterozygous state is both extremely ephemeral and produces a phenotype that is, in fact, not intermediate between the two endpoint states.

Our study suggests that the continuity between states in such transitions could be more limited than previously expected. For instance, we recognize the idiosyncrasy of our system and conditions, both in terms of its relative simplicity and the unique traits associated with *S. cerevisiae*; however, we feel that this serves as a proof-of-concept model demonstrating the potential for similar evolutionary dynamics in natural populations.

### (c) Evolutionary mechanisms for innovation

Multicellular snowflake phenotypes evolved in as little as 7 days in the original experiment [6]. Moreover, we observed the evolution of the snowflake phenotypes within 2–3 days in 50% of the initially heterozygous populations. We tested populations both when put through our settling selection regime and when being propagated under standard batch culture conditions. Results suggest a rapid and random LOH towards the homozygous recessive state. While LOH may also occur with equal frequency towards the homozygous dominant state, the phenotypes for these two genotypes were not readily discernable and thus were not measured as part of this study.

LOH events are common and can happen over short evolutionary timescales in yeast [42,43]. High rates of LOH play an important role in the expression of recessive mutations within diploid microbial eukaryotes, particularly in organisms for which sexual reproduction is rare or absent [17,44]. In *S. cerevisiae*, rates of LOH in some parts of the genome can be much higher than mutation rates [45], and recent studies show that initial heterozygosity does not influence the rate or mechanisms of LOH [46]. LOH events, a source of adaptive genetic variation, however, have largely been overlooked as they are often associated with DNA damage and the exposure of deleterious recessive mutations [46].

Even though we did not identify the exact mechanism by which LOH affects this locus, the ace2 alleles identified in evolved multicellular strains that carried this mutation were identical [27]. In principle, cryptic meiosis could generate diploid homozygous multicellular individuals in our selected populations. But meiosis in yeast rarely occurs in rich media [47], providing little opportunity for meiosis to produce multicellular homozygotes. The fact that multicellular phenotypes evolve even under non-selective conditions suggests a high rate of LOH events, which is inconsistent with cryptic meiosis, especially because multicellularity itself incurs substantial growth rate reductions. Interallelic gene conversion might have accelerated the spread of ace2 advantageous mutations, fixing a recessive mutation under settling selection despite any deleterious effects from heterozygosity. Previous theoretical studies have described the evolutionary implications of gene conversion in speeding adaptation and helping circumvent low fitness valleys caused by deleterious intermediate steps [48,49].

## 5. Conclusion

We investigated the basis for adaptive evolution by reconstructing the evolutionary pathway from uni- to multicellularity in experimental populations of diploid yeast. Despite arising due to selection for settling rates (a proxy for size), the evolution of multicellularity involves a decrease in individual size during the very first evolutionary steps. Two LOF alleles at the ACE2 locus are necessary to confer multicellularity, which results in anapproximately 200% increase in individual size relative to the WT. Heterozygous genotypes, however, are 20% smaller than the WT. Nevertheless, populations of heterozygotes give rise to multicellular genotypes more readily than unicellular genotypes with two functional alleles through LOH. Thus, the evolutionary transition to multicellularity in our system involves size reduction prior to accessing adaptive increases in size, resulting in discordance between the phenotypic and genotypic transitions.

We highlight the importance of assessing intermediate steps involving both genotypic and phenotypic aspects of complexity in adaptations that might require descending into fitness valleys due to underdominance. This study demonstrated causal relationships linking phenotype to population fitness and mechanisms by which evolutionary changeover might be promoted. The lack of a predictive framework that recognizes this hinders knowledge of how organization and heritable information scale across phenotypic and genetic adaptations, especially in the evolution of more complex derived forms. Similar studies are necessary to understand the evolutionary dynamics and their implications on patterns of diversity among organisms, populations and species.

## Data Availability

All raw data presented and discussed in this paper can be found in the Dryad Digital Repository [50]. The data are provided in electronic supplementary material [51].
